# Crystal structure of *rac*-3,9-bis­(2,6-di­fluoro­phen­yl)-2,4,8,10-tetra­oxa­spiro[5.5]undeca­ne

**DOI:** 10.1107/S2056989015001206

**Published:** 2015-01-24

**Authors:** Liang Chen, Zhengyi Li, Linlin Jin, Xiaoqiang Sun, Zhiming Wang

**Affiliations:** a Advanced Catalysis and Green Manufacturing Collaborative Innovation Center, Changzhou University, Changzhou 213164, Jiangsu, People’s Republic of China; b Key Laboratory of Fine Chemical Engineering, Changzhou University, Changzhou, 213164, Jiangsu, People’s Republic of China

**Keywords:** crystal structure, oxo-spiro­cyclic, helical hydrogen-bonded chains, axial chirality

## Abstract

The title compound, C_19_H_16_F_4_O_4_, was prepared by the condensation reaction of 2,6-di­fluoro­benzaldehyde and penta­erythritol. The whole mol­ecule is generated by twofold rotational symmetry. The two six-membered O-heterocycles adopt chair conformations through a shared spiro-carbon atom that is located on the crystallographic twofold rotation axis. In this conformation, the two aromatic rings are located at the equatorial positions of the O-heterocycles. The conformation of this doubly substituted tetra­oxa­spiro system is chiral. In the crystal, mol­ecules are linked by C—H⋯O hydrogen bonds, forming layers parallel to (100). These layers are linked by C—H⋯F hydrogen bonds into a three-dimensional structure.

## Related literature   

For the use of tetra­oxa­spiro­[5.5]undeca­nes, see: Cismaş *et al.* (2005 ▸); Sondhi *et al.* (2009 ▸); Sauriat-Dorizon *et al.* (2003 ▸). For chiral conformations of tetra­oxa­spiro­[5.5]undeca­nes, see: Mihiş *et al.* (2008 ▸). For opposite enanti­omers of tetra­oxa­spiro­[5.5]undeca­nes, see: Sun *et al.* (2010 ▸).
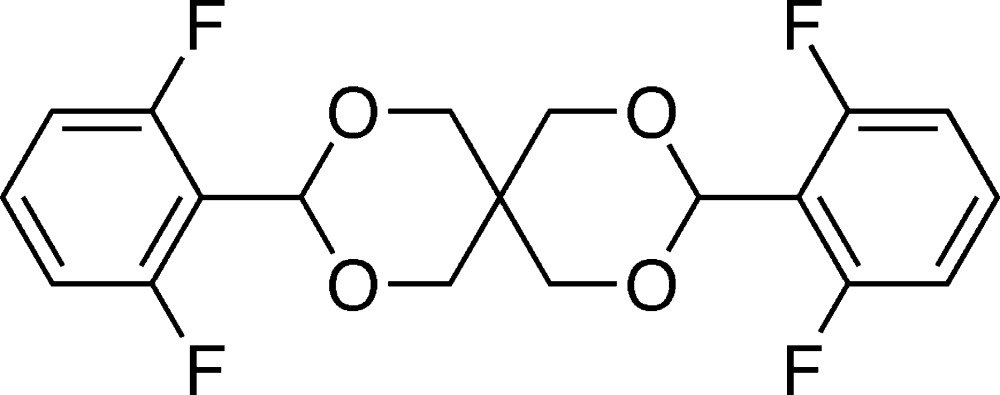


## Experimental   

### Crystal data  

C_19_H_16_F_4_O_4_
*M* *_r_* = 384.32
Monoclinic, 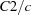

*a* = 28.960 (5) Å
*b* = 5.5627 (11) Å
*c* = 11.205 (2) Å
β = 95.442 (4)°
*V* = 1797.0 (6) Å^3^
*Z* = 4
Mo *K*α radiation
μ = 0.13 mm^−1^
*T* = 296 K
0.20 × 0.18 × 0.15 mm

### Data collection  

Bruker APEXII CCD area-detector diffractometer
Absorption correction: multi-scan (*SADABS*; Bruker, 2009 ▸) *T*_min_ = 0.975, *T*_max_ = 0.981
4856 measured reflections
1671 independent reflections
1444 reflections with *I* > 2σ(*I*)
*R*_int_ = 0.031

### Refinement  

*R*[*F* ^2^ > 2σ(*F* ^2^)] = 0.050
*wR*(*F* ^2^) = 0.148
*S* = 1.01
1671 reflections
124 parameters
H-atom parameters constrained
Δρ_max_ = 0.26 e Å^−3^
Δρ_min_ = −0.34 e Å^−3^

### 

Data collection: *APEX2* (Bruker, 2009 ▸); cell refinement: *SAINT* (Bruker, 2009 ▸); data reduction: *SAINT*; program(s) used to solve structure: *SHELXS97* (Sheldrick, 2008 ▸); program(s) used to refine structure: *SHELXL97* (Sheldrick, 2015 ▸); molecular graphics: *SHELXTL* (Sheldrick, 2008 ▸); software used to prepare material for publication: *SHELXTL*.

## Supplementary Material

Crystal structure: contains datablock(s) I. DOI: 10.1107/S2056989015001206/su5070sup1.cif

Structure factors: contains datablock(s) I. DOI: 10.1107/S2056989015001206/su5070Isup2.hkl

Supporting information file. DOI: 10.1107/S2056989015001206/su5070Isup3.cml

**Fig. 1.**
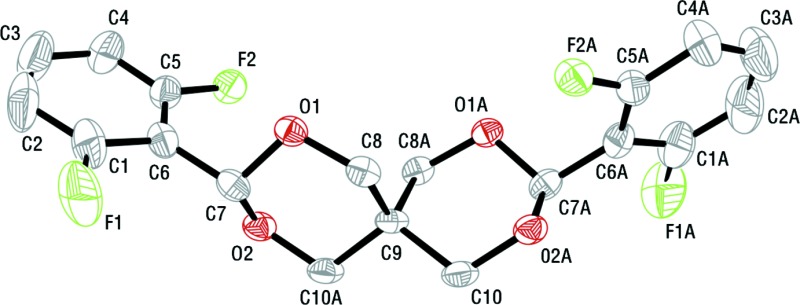
The molecular structure of the title compound, with atom labelling. Displacement ellipsoids are drawn at the 30% probability level.

. DOI: 10.1107/S2056989015001206/su5070fig1.tif
The mol­ecular structure of the title compound, with atom labelling. Displacement ellipsoids are drawn at the 30% probability level.

**Fig. 2.**
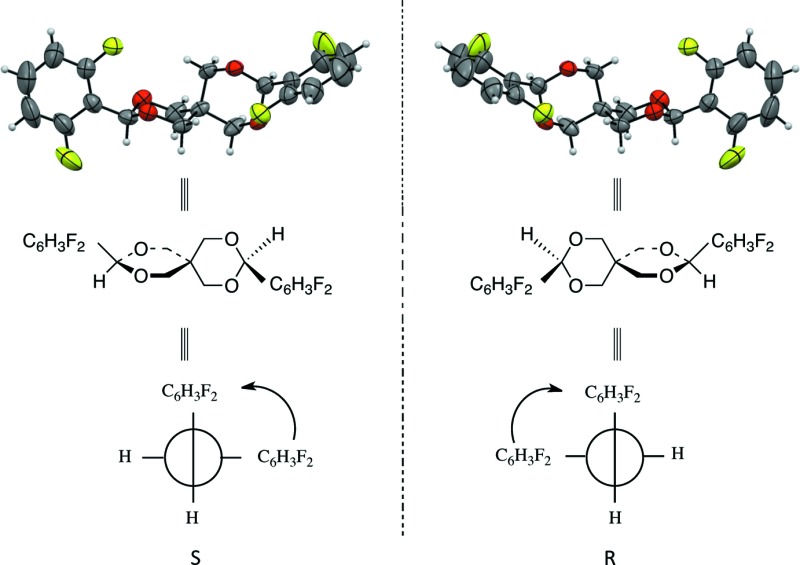
The crystal structure of enantiomers of the title compound, showing the two opposite enantiomers.

**Fig. 3.**
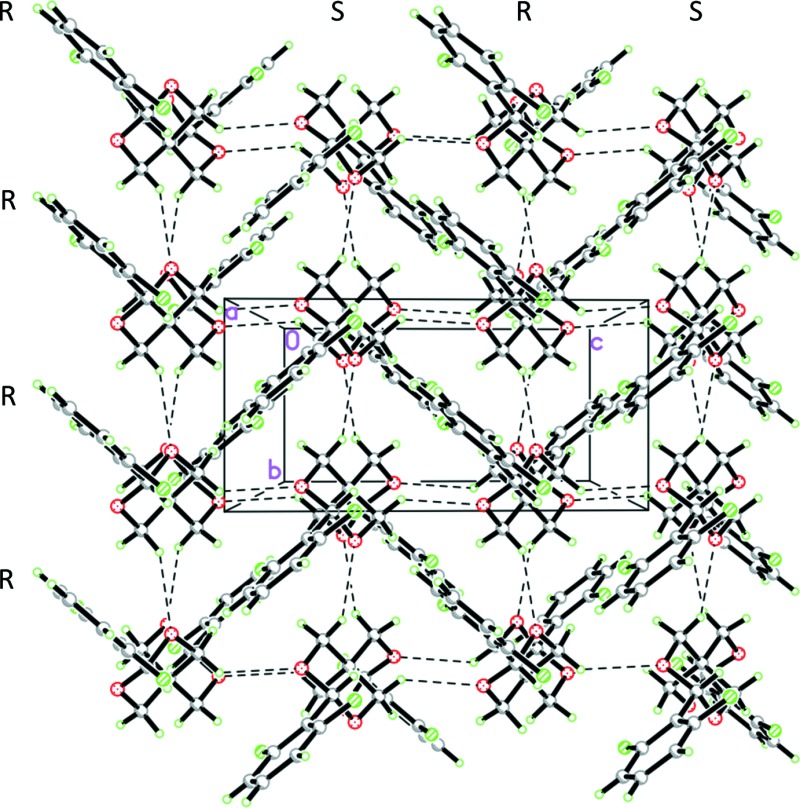
Part of the layered crystal structure of the title compound, showing the weak C—H···O interactions between the molecules. The same enantiomers are linked along the b axis, and the different enantiomers are linked alternatively along the c axis.

**Fig. 4.**
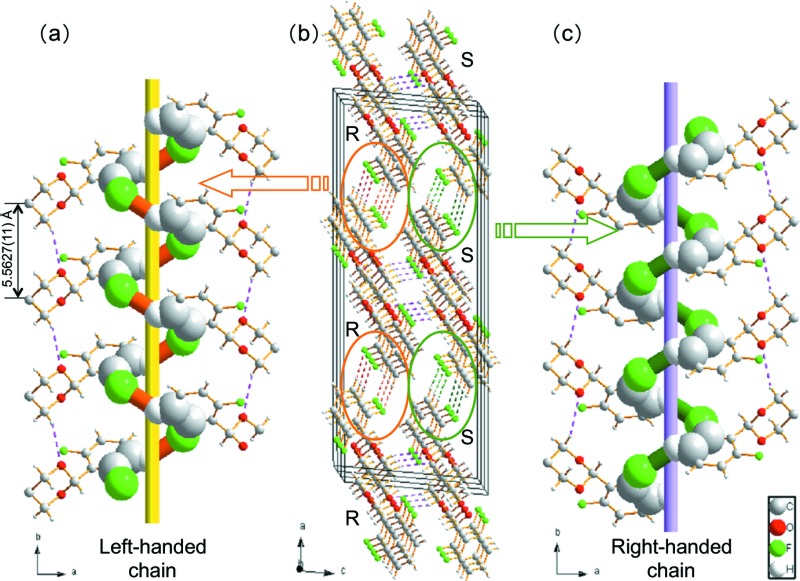
A view of a part of the crystal structure of the title compound: (a) Left-handed helical chains of molecules with R configuration connected via the weak C—H···F interactions (represented with the orange dashed lines) along the b axis; (b) the weak C—H···F interactions of the left-handed helical chains (indicated as the orange dashed line in the orange oval rings), the weak C—H···F interactions of the right-handed helical chains (represented as the green dashed line in the green oval rings); and the weak C—H···O interactions (represented as the red dashed lines); (c) Right-handed helical chains of the molecules with S configuration connected via the weak C—H···F interactions (represented with the green dashed lines) along the b axis.

. DOI: 10.1107/S2056989015001206/su5070fig2.tif
The crystal structure of enanti­omers of the title compound, showing the two opposite enanti­omers.

b c . DOI: 10.1107/S2056989015001206/su5070fig3.tif
Part of the layered crystal structure of the title compound, showing the weak C—H⋯O inter­actions between the mol­ecules. The same enanti­omers are linked along the *b* axis, and the different enanti­omers are linked alternatively along the *c* axis.

a R via b b c via b . DOI: 10.1107/S2056989015001206/su5070fig4.tif
A view of a part of the crystal structure of the title compound: (*a*) Left-handed helical chains of mol­ecules with *R* configuration connected *via* the weak C—H⋯F inter­actions (represented with the orange dashed lines) along the *b* axis; (*b*) the weak C—H⋯F inter­actions of the left-handed helical chains (indicated as the orange dashed line in the orange oval rings), the weak C—H⋯F inter­actions of the right-handed helical chains (represented as the green dashed line in the green oval rings); and the weak C—H⋯O inter­actions (represented as the red dashed lines); (*c*) Right-handed helical chains of the mol­ecules with S configuration connected *via* the weak C—H⋯F inter­actions (represented with the green dashed lines) along the *b* axis.

CCDC reference: 859906

Additional supporting information:  crystallographic information; 3D view; checkCIF report

## Figures and Tables

**Table 1:** Hydrogen-bond geometry (, )

*D*H*A*	*D*H	H*A*	*D* *A*	*D*H*A*
C8H8*A*O2^i^	0.97	2.57	3.334(2)	136
C10H10*B*O1^ii^	0.97	2.56	3.410(2)	146
C2H2F1^iii^	0.93	2.56	3.351(3)	143

Symmetry codes: (i) 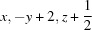; (ii) 
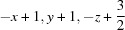
; (iii) 
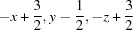
.

## supplementary crystallographic information

### S1. Comment

Tetra­oxa­spiro­[5.5]undecanes derived from penta­erythritol are a class of the oxo-spiro­cyclic compounds. 3,9-disubstituted spiro­cyclic compounds have inter­esting stereochemical properties like axial chirality, and play important roles in the fields like, pesticide chemistry (Cismaş *et al.*, 2005), medicinal chemistry (Sondhi *et al.*, 2009), and materials chemistry (Sauriat-Dorizon *et al.*, 2003). Due to their stereochemical properties (Mihiş *et al.*, 2008), these compounds in the solid state always adopt chiral conformations, and are built from opposite enanti­omers (Sun *et al.*, 2010), as shown below.

In the title compound, (I), the molecule is consisted of the identical two components though a shared spiro-C9 atom with a crystallographic two-fold symmetry axis (Fig. 1). The two six-membered heterocycles both adopt chair conformation. And the two aromatic residues both are located in the equatorial positions (atom C7) of the tetra-oxa­spiro skeleton, through the staggered structure with the six-membered heterocycles, with the torsion angle O1—C7—C6—C1 of -112.5 (2) °.

The phenyl rings, as groups are much larger than hydrogen atoms, are located at the equatorial positions (atom C7) of six-membered O-heterocycles, while the H7 atoms are placed at the axial positions. In the oxo-spiro­cyclic skeleton, the distance between atoms O1 and O1A is longer than the distance between atoms O2 and O2A, with difference in value of ca. 1.115 Å. The molecule looks like a two-bladed propeller with the dihedral angle between the mean planes of (C1–C6) and (C1A–C6A) equal to 82.9 (5) °. Two opposite enanti­omers, with equal numbers, are present in the crystal (Fig. 2) with a centrosymmetric space group (C2/c).

The packing of molecules of (I), is determined by the presence of numerous weak inter­molecular inter­actions (Table 1). The C10—H10B···O1 inter­actions link the same molecules with the identical configurations R or S, respectively, into parallel chains along the b axis, furthermore, the C8—H8A···O2 inter­actions link the R and S chains alternatively along the c axis into a two-dimensional layered reticulate structure (Fig. 3) in bc plane. The donors and acceptors of the C—H···O inter­actions are barely constituted by the C and O atoms from the oxo-spiro­cyclic skeleton.

In crystalline states, those achiral layered structures are stacked into a three-dimensional structure by weak C—H···F inter­molecular inter­actions between the same enanti­omers (Fig. 4 and Table 1). It is worth mentioning that the C—H···F inter­actions constitute left/right-handed homochiral helical chains along the b axis (Fig. 4), with left-handed helical chains of the molecules of R configuration (Fig. 4a), and right-handed helical chains of S conformation molecules (Fig. 4c). And, we can clearly see that each helix in the homochiral chains consists of two molecules, with a pitch of 5.5627 (11) A (Fig. 4a). The two kinds of helical chains are arranged parallel to each other in the vertical direction of bc plan, with the same-handed chains along the a axis, and the different-handed chains along the c axis alternatively (Fig. 4b).

### S2. Experimental

A catalytic qu­antity (0.04, 0.2 mmol) of p-toluene­sulfonic acid was added to a solution of 2,6-di­fluoro­benzaldehyde (1.14 g, 8 mmol) and penta­erythritol (0.52 mg, 3.8 mmol) in toluene (40 ml), and this mixture was then heated under reflux for 4 h in a flask equipped with a condenser and a water trap. The solution was washed with the sodium carbonate solution (10 %). Then, the organic layer was separated and the solvent was evaporated under the vacuum conditions and the residue was dried. The resulting solid product, (yield: 80%; m.p.: 509.5 - 510.2 K), was recrystallized from ethanol. The colourless crystals suitable for single-crystal X-ray diffraction, were also grown from ethanol. ^1^H NMR (300 MHz, DMSO-D6): δ 3.68–3.81(m, 4H), 3.94–3.98 (d, 2H), 4.66–4.69 (d, 2H), 5.89 (s, 2H), 7.11–7.17 (t, 4H), 7.46–7.53 (m, 2H).

### S3. Refinement

All the H atoms were placed in calculated positions and allowed to ride on their parent atoms: C—H = 0.93 - 0.98 Å with U_iso_(H) = 1.2U_eq_(C).

### Crystal data

**Table:**

C_19_H_16_F_4_O_4_	*F*(000) = 792
*M**_r_* = 384.32	*D*_x_ = 1.421 Mg m^−^^3^
Monoclinic, *C*2/*c*	Mo *K*α radiation, λ = 0.71073 Å
Hall symbol: -C 2yc	Cell parameters from 2905 reflections
*a* = 28.960 (5) Å	θ = 2.8–29.7°
*b* = 5.5627 (11) Å	µ = 0.13 mm^−^^1^
*c* = 11.205 (2) Å	*T* = 296 K
β = 95.442 (4)°	Block, colourless
*V* = 1797.0 (6) Å^3^	0.20 × 0.18 × 0.15 mm
*Z* = 4	

### Data collection

**Table:**

Bruker APEXII CCD area-detector diffractometer	1671 independent reflections
Radiation source: fine-focus sealed tube	1444 reflections with *I* > 2σ(*I*)
Graphite monochromator	*R*_int_ = 0.031
phi and ω scans	θ_max_ = 25.5°, θ_min_ = 1.4°
Absorption correction: multi-scan (*SADABS*; Bruker, 2009)	*h* = −34→34
*T*_min_ = 0.975, *T*_max_ = 0.981	*k* = −6→6
4856 measured reflections	*l* = −13→11

### Refinement

**Table:**

Refinement on *F*^2^	Secondary atom site location: difference Fourier map
Least-squares matrix: full	Hydrogen site location: inferred from neighbouring sites
*R*[*F*^2^ > 2σ(*F*^2^)] = 0.050	H-atom parameters constrained
*wR*(*F*^2^) = 0.148	*w* = 1/[σ^2^(*F*_o_^2^) + (0.0997*P*)^2^ + 0.5847*P*] where *P* = (*F*_o_^2^ + 2*F*_c_^2^)/3
*S* = 1.00	(Δ/σ)_max_ = 0.001
1671 reflections	Δρ_max_ = 0.26 e Å^−^^3^
124 parameters	Δρ_min_ = −0.34 e Å^−^^3^
0 restraints	Extinction correction: *SHELXL*, Fc^*^=kFc[1+0.001xFc^2^λ^3^/sin(2θ)]^-1/4^
Primary atom site location: structure-invariant direct methods	Extinction coefficient: 0.057 (5)

### Special details

**Table:**

Geometry. All e.s.d.'s (except the e.s.d. in the dihedral angle between two l.s. planes) are estimated using the full covariance matrix. The cell e.s.d.'s are taken into account individually in the estimation of e.s.d.'s in distances, angles and torsion angles; correlations between e.s.d.'s in cell parameters are only used when they are defined by crystal symmetry. An approximate (isotropic) treatment of cell e.s.d.'s is used for estimating e.s.d.'s involving l.s. planes.
Refinement. Refinement of *F*^2^ against ALL reflections. The weighted *R*-factor *wR* and goodness of fit *S* are based on *F*^2^, conventional *R*-factors *R* are based on *F*, with *F* set to zero for negative *F*^2^. The threshold expression of *F*^2^ > σ(*F*^2^) is used only for calculating *R*-factors(gt) *etc*. and is not relevant to the choice of reflections for refinement. *R*-factors based on *F*^2^ are statistically about twice as large as those based on *F*, and *R*- factors based on ALL data will be even larger.

### Fractional atomic coordinates and isotropic or equivalent isotropic displacement parameters (Å^2^)

**Table:**

	*x*	*y*	*z*	*U*_iso_*/*U*_eq_	
C9	0.5000	1.0314 (3)	0.7500	0.0444 (5)	
O2	0.56492 (4)	1.05258 (19)	0.62616 (10)	0.0529 (4)	
O1	0.56527 (4)	0.75364 (18)	0.77199 (9)	0.0478 (4)	
F2	0.57010 (3)	0.5988 (2)	0.51818 (9)	0.0650 (4)	
C8	0.53125 (5)	0.8761 (3)	0.83549 (13)	0.0469 (4)	
H8A	0.5468	0.9763	0.8978	0.056*	
H8B	0.5126	0.7589	0.8734	0.056*	
C7	0.59217 (6)	0.9188 (3)	0.71277 (14)	0.0516 (4)	
H7	0.6082	1.0281	0.7716	0.062*	
C10	0.46902 (7)	1.1913 (3)	0.81939 (15)	0.0569 (5)	
H10A	0.4880	1.2783	0.8810	0.068*	
H10B	0.4535	1.3082	0.7652	0.068*	
C6	0.62720 (6)	0.7791 (3)	0.65080 (16)	0.0587 (5)	
C5	0.61516 (6)	0.6237 (3)	0.55644 (18)	0.0614 (5)	
C4	0.64657 (9)	0.4916 (5)	0.4995 (3)	0.0962 (8)	
H4	0.6369	0.3890	0.4365	0.115*	
C1	0.67413 (8)	0.7922 (6)	0.6860 (3)	0.0917 (8)	
F1	0.68770 (5)	0.9389 (5)	0.78045 (18)	0.1351 (8)	
C2	0.70735 (8)	0.6647 (9)	0.6309 (4)	0.1288 (13)	
H2	0.7387	0.6806	0.6563	0.155*	
C3	0.69284 (12)	0.5162 (8)	0.5389 (4)	0.1302 (13)	
H3	0.7147	0.4287	0.5015	0.156*	

### Atomic displacement parameters (Å^2^)

**Table:**

	*U*^11^	*U*^22^	*U*^33^	*U*^12^	*U*^13^	*U*^23^
C9	0.0647 (13)	0.0304 (9)	0.0386 (10)	0.000	0.0074 (9)	0.000
O2	0.0676 (8)	0.0444 (6)	0.0482 (7)	−0.0015 (5)	0.0131 (5)	0.0078 (5)
O1	0.0523 (6)	0.0447 (6)	0.0457 (6)	−0.0014 (4)	0.0011 (4)	0.0065 (4)
F2	0.0611 (7)	0.0655 (7)	0.0669 (7)	−0.0013 (4)	−0.0020 (5)	−0.0138 (5)
C8	0.0595 (9)	0.0458 (8)	0.0350 (7)	−0.0063 (6)	0.0023 (6)	0.0012 (6)
C7	0.0543 (9)	0.0527 (9)	0.0464 (8)	−0.0136 (7)	−0.0020 (7)	−0.0004 (6)
C10	0.0834 (12)	0.0336 (8)	0.0560 (10)	0.0014 (7)	0.0177 (8)	−0.0043 (6)
C6	0.0482 (9)	0.0680 (11)	0.0594 (10)	−0.0062 (7)	0.0029 (7)	0.0097 (8)
C5	0.0591 (10)	0.0624 (11)	0.0640 (11)	0.0034 (8)	0.0114 (8)	0.0034 (8)
C4	0.0832 (15)	0.1023 (18)	0.1070 (19)	0.0196 (14)	0.0290 (14)	−0.0103 (16)
C1	0.0510 (11)	0.124 (2)	0.0972 (17)	−0.0138 (12)	−0.0083 (10)	0.0036 (15)
F1	0.0684 (9)	0.195 (2)	0.1355 (14)	−0.0375 (11)	−0.0249 (9)	−0.0258 (13)
C2	0.0455 (12)	0.183 (4)	0.158 (3)	0.0131 (17)	0.0088 (15)	0.019 (3)
C3	0.0830 (19)	0.149 (3)	0.164 (3)	0.038 (2)	0.042 (2)	−0.001 (3)

### Geometric parameters (Å, º)

**Table:**

C9—C8^i^	1.5226 (19)	C10—O2^i^	1.4309 (19)
C9—C8	1.5227 (18)	C10—H10A	0.9700
C9—C10	1.5277 (19)	C10—H10B	0.9700
C9—C10^i^	1.5277 (19)	C6—C1	1.381 (3)
O2—C7	1.405 (2)	C6—C5	1.384 (3)
O2—C10^i^	1.4309 (19)	C5—C4	1.373 (3)
O1—C7	1.4106 (18)	C4—C3	1.377 (5)
O1—C8	1.4400 (18)	C4—H4	0.9300
F2—C5	1.342 (2)	C1—F1	1.364 (3)
C8—H8A	0.9700	C1—C2	1.386 (5)
C8—H8B	0.9700	C2—C3	1.356 (6)
C7—C6	1.500 (2)	C2—H2	0.9300
C7—H7	0.9800	C3—H3	0.9300
			
C8^i^—C9—C8	110.87 (16)	C9—C10—H10A	109.4
C8^i^—C9—C10	107.93 (9)	O2^i^—C10—H10B	109.4
C8—C9—C10	110.67 (9)	C9—C10—H10B	109.4
C8^i^—C9—C10^i^	110.67 (9)	H10A—C10—H10B	108.0
C8—C9—C10^i^	107.93 (9)	C1—C6—C5	114.9 (2)
C10—C9—C10^i^	108.76 (16)	C1—C6—C7	122.08 (19)
C7—O2—C10^i^	110.78 (12)	C5—C6—C7	123.01 (15)
C7—O1—C8	111.00 (11)	F2—C5—C4	117.6 (2)
O1—C8—C9	110.56 (10)	F2—C5—C6	118.38 (15)
O1—C8—H8A	109.5	C4—C5—C6	124.0 (2)
C9—C8—H8A	109.5	C5—C4—C3	117.7 (3)
O1—C8—H8B	109.5	C5—C4—H4	121.1
C9—C8—H8B	109.5	C3—C4—H4	121.1
H8A—C8—H8B	108.1	F1—C1—C6	117.2 (2)
O2—C7—O1	111.78 (12)	F1—C1—C2	119.4 (2)
O2—C7—C6	108.36 (13)	C6—C1—C2	123.5 (3)
O1—C7—C6	107.94 (13)	C3—C2—C1	118.2 (2)
O2—C7—H7	109.6	C3—C2—H2	120.9
O1—C7—H7	109.6	C1—C2—H2	120.9
C6—C7—H7	109.6	C2—C3—C4	121.8 (3)
O2^i^—C10—C9	111.29 (12)	C2—C3—H3	119.1
O2^i^—C10—H10A	109.4	C4—C3—H3	119.1
			
C7—O1—C8—C9	57.50 (15)	C1—C6—C5—F2	179.13 (19)
C8^i^—C9—C8—O1	69.20 (9)	C7—C6—C5—F2	0.6 (3)
C10—C9—C8—O1	−171.06 (11)	C1—C6—C5—C4	−0.5 (3)
C10^i^—C9—C8—O1	−52.16 (15)	C7—C6—C5—C4	−179.1 (2)
C10^i^—O2—C7—O1	61.60 (16)	F2—C5—C4—C3	−179.8 (3)
C10^i^—O2—C7—C6	−179.58 (12)	C6—C5—C4—C3	−0.1 (4)
C8—O1—C7—O2	−61.92 (15)	C5—C6—C1—F1	−178.6 (2)
C8—O1—C7—C6	179.00 (11)	C7—C6—C1—F1	0.0 (3)
C8^i^—C9—C10—O2^i^	52.40 (16)	C5—C6—C1—C2	1.2 (4)
C8—C9—C10—O2^i^	−69.09 (16)	C7—C6—C1—C2	179.8 (3)
C10^i^—C9—C10—O2^i^	172.51 (17)	F1—C1—C2—C3	178.6 (4)
O2—C7—C6—C1	126.2 (2)	C6—C1—C2—C3	−1.2 (5)
O1—C7—C6—C1	−112.5 (2)	C1—C2—C3—C4	0.5 (6)
O2—C7—C6—C5	−55.3 (2)	C5—C4—C3—C2	0.2 (6)
O1—C7—C6—C5	65.9 (2)		

Symmetry code: (i) −*x*+1, *y*, −*z*+3/2.

### Hydrogen-bond geometry (Å, º)

**Table:**

*D*—H···*A*	*D*—H	H···*A*	*D*···*A*	*D*—H···*A*
C8—H8*A*···O2^ii^	0.97	2.57	3.334 (2)	136
C10—H10*B*···O1^iii^	0.97	2.56	3.410 (2)	146
C2—H2···F1^iv^	0.93	2.56	3.351 (3)	143

Symmetry codes: (ii) *x*, −*y*+2, *z*+1/2; (iii) −*x*+1, *y*+1, −*z*+3/2; (iv) −*x*+3/2, *y*−1/2, −*z*+3/2.
