# Supplementary figures and images for: Crystal structure of rac-3,9-bis­(2,6-di­fluoro­phen­yl)-2,4,8,10-tetra­oxa­spiro[5.5]undeca­ne
Source: Acta Crystallogr E Crystallogr Commun. 2015 Jan 24;71(Pt 2):o127–8. doi: 10.1107/S2056989015001206 (PMC4384547; doi:10.1107/S2056989015001206)

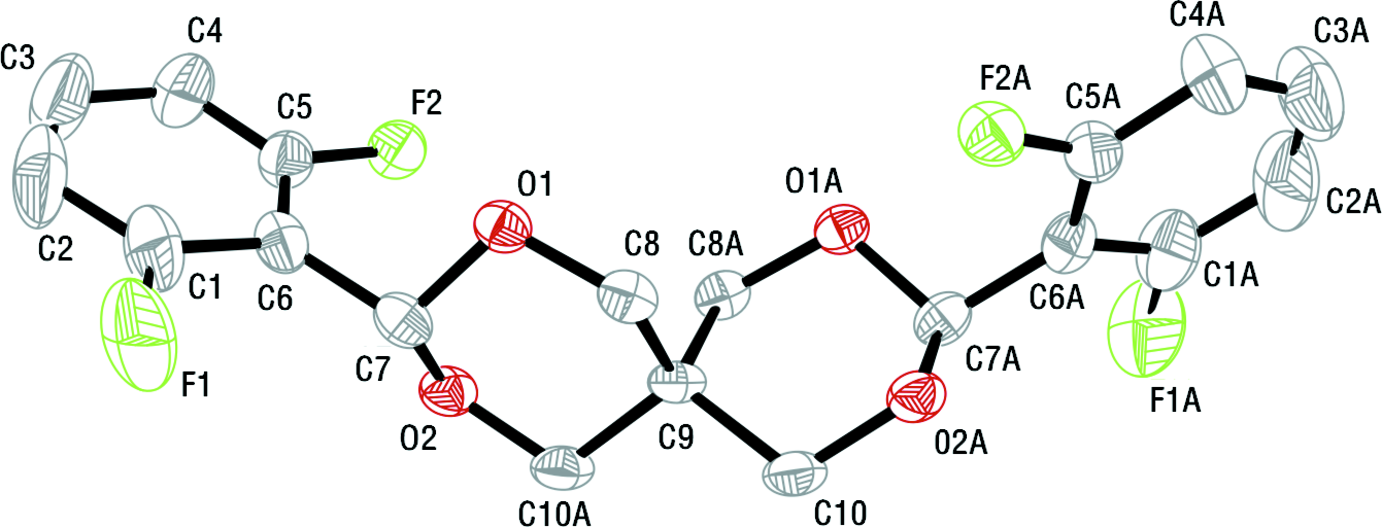

Supplement: Supplementary file 4 [file e-71-0o127-fig1.tif]

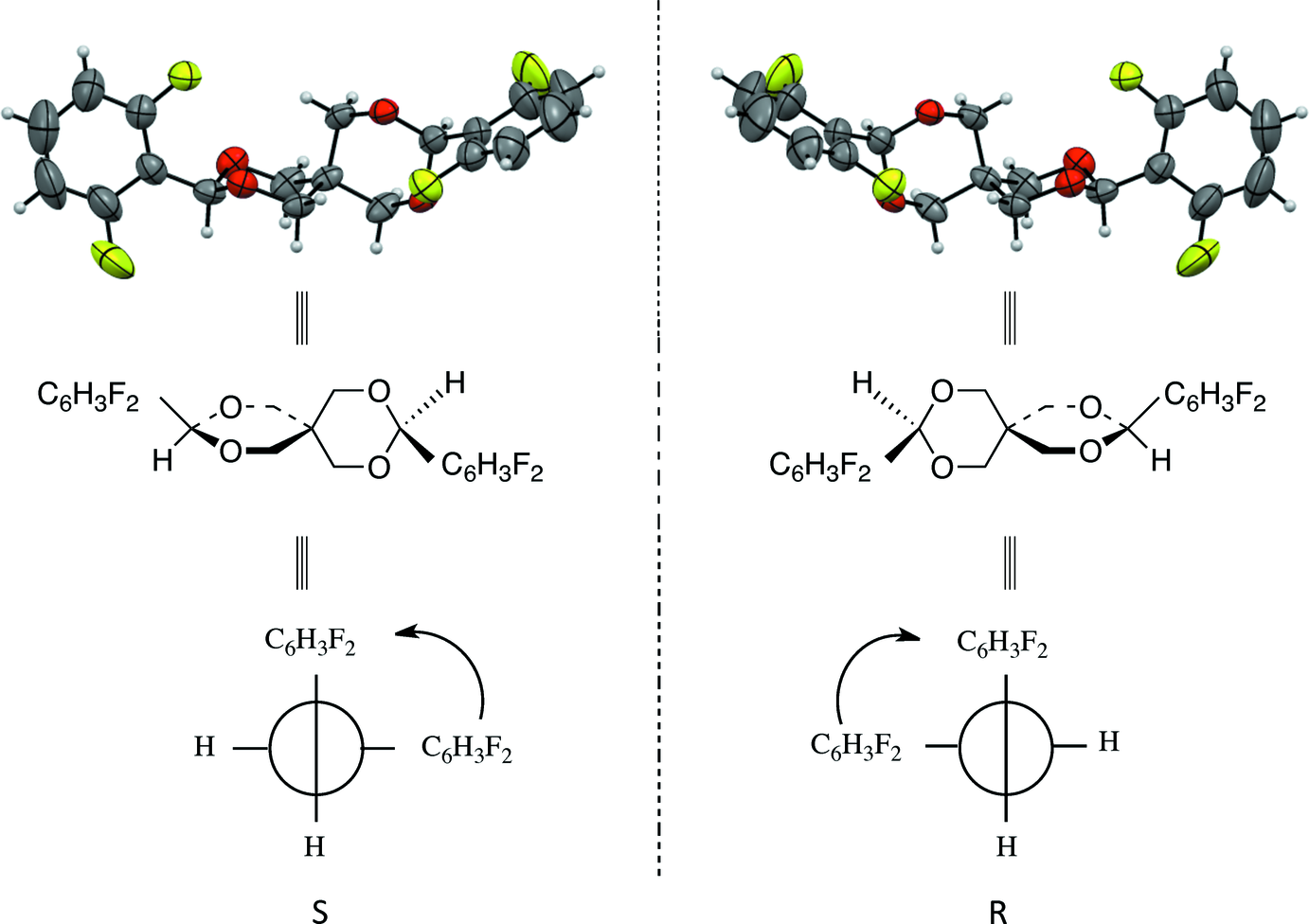

Supplement: Supplementary file 5 [file e-71-0o127-fig2.tif]

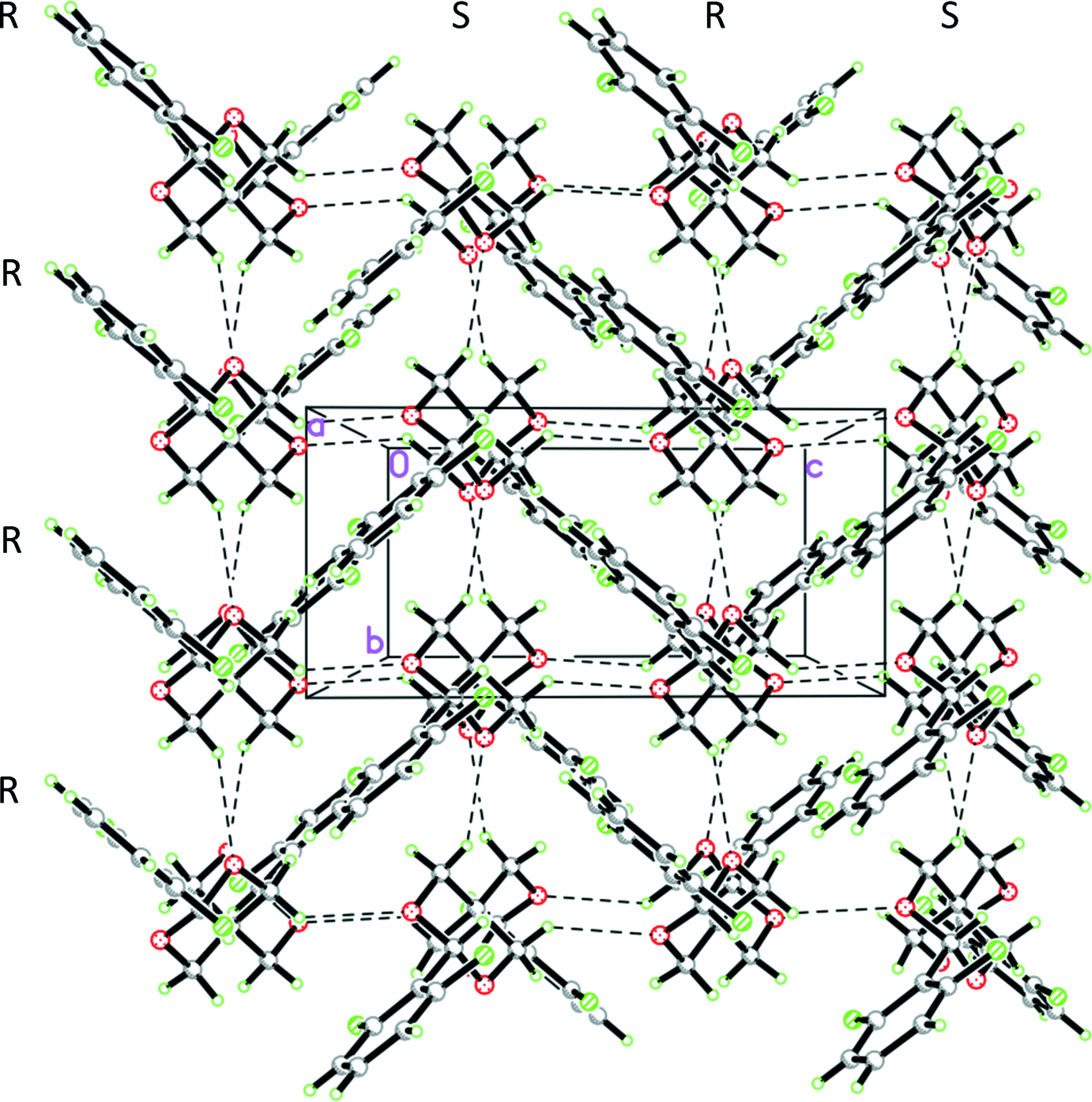

Supplement: Supplementary file 6 [file e-71-0o127-fig3.tif]

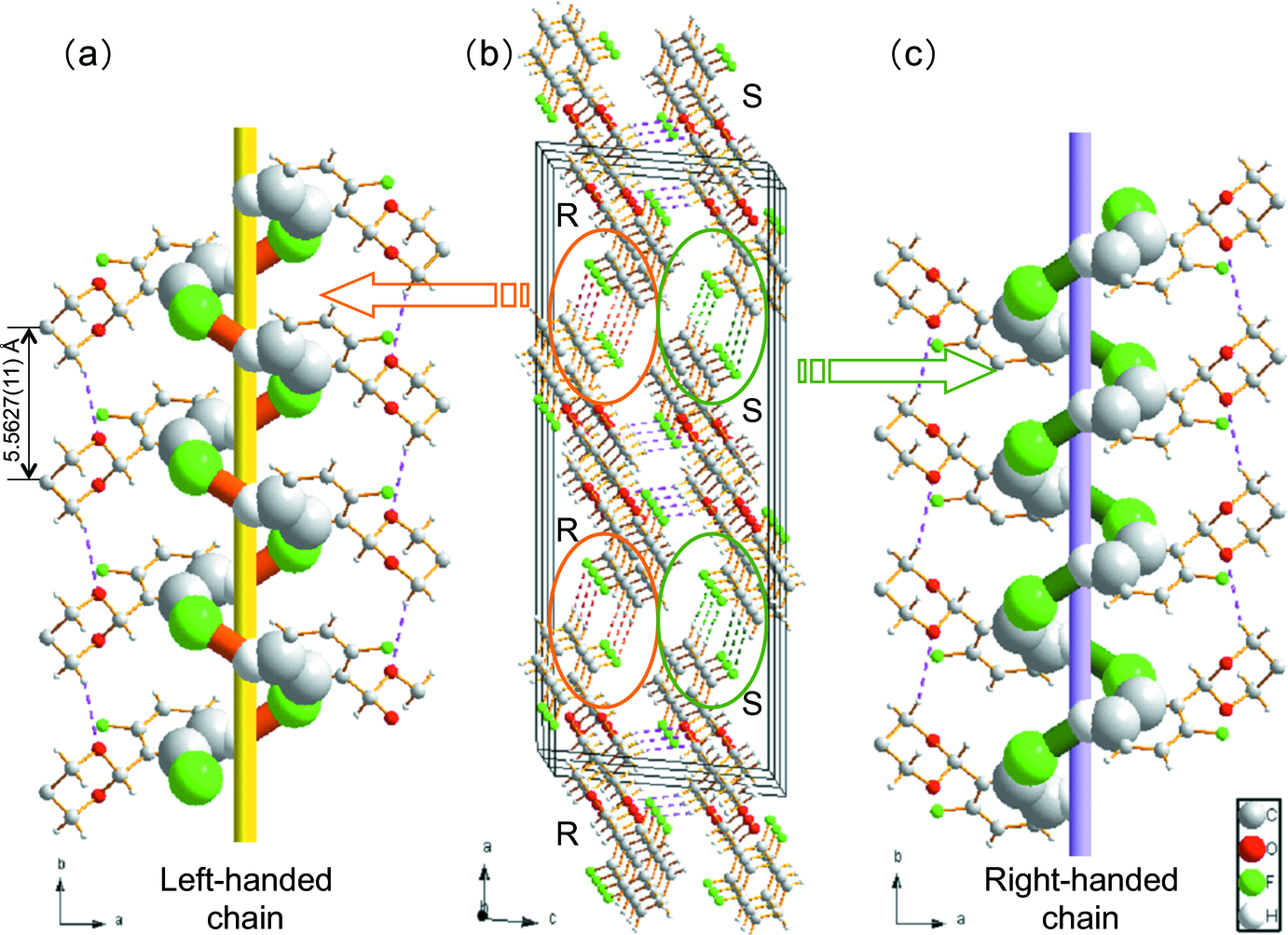

Supplement: Supplementary file 7 [file e-71-0o127-fig4.tif]
